# Zoonoses: a potential obstacle to the growing wildlife industry of Namibia

**DOI:** 10.3402/iee.v2i0.18365

**Published:** 2012-10-15

**Authors:** Kudakwashe Magwedere, Maria Y. Hemberger, Louw C. Hoffman, Francis Dziva

**Affiliations:** 1 Department of Animal Sciences, Stellenbosch University, Stellenbosch, South Africa; 2 Division of Veterinary Public Health, Directorate of Veterinary Services, Mariental, Namibia; 3 Division of Microbiology, Institute for Animal Health, Compton, United Kingdom

**Keywords:** Zoonoses, wildlife, livestock, public health, meat safety, Namibia

## Abstract

Zoonoses, which account for approximately 75% of emerging human infectious diseases worldwide, pose a re-emerging threat to public health. With an ever-increasing interrelationship between humans, livestock and wildlife species, the threat to human health will rise to unprecedented levels. Wildlife species contribute to the majority of emerging diseases; therefore, there is an urgent need to define control systems of zoonoses of wildlife origin but very little information exists. In this review, we examine prevalent zoonotic infections reported in Namibia between 1990 and 2009 and assess their potential impact on the growing wildlife industry. A wide spectrum of zoonotic diseases was confirmed in both livestock and wildlife species, with rabies and anthrax cases being over-represented and also showing the widest species distribution. Whilst vaccination and ante-mortem inspection against these diseases may curb infected livestock species from entering the human food chain, such practices are difficult to implement in free-ranging wildlife species. In this context, there is a need to improve existing control measures and/or develop novel and better interventional strategies to reduce the threat of this re-emerging global problem. This review provides the basis for initiating a multidisciplinary evidence-based approach to control zoonoses in countries with thriving wildlife and game farming.

The global trade in wildlife has historically contributed to the emergence and spread of infectious diseases due to new host adaptation by pathogens, increased human susceptibility to disease, changing environments, intensification of the human–animal interface, movement of humans and animals across international borders, emerging anti-microbial resistance, and minimal pathogen surveillance that has precluded assessment of the health risks (1–5). Zoonotic diseases are transmitted from vertebrate animals to humans through direct or indirect exposure to live animals, their by-products or contaminated environments (6, 7). Although the conversion of wildlife habitats into arable land for crops, pastures and lodging camps can be viewed as the biggest driver of emerging and re-emerging zoonotic diseases associated with wildlife species, it is the ever increasing wildlife–human–domestic animal interface, including the consumption of game meat around the world that attracts recent concerns and challenges (5). Traditionally, most meat-borne disease outbreaks arise from improper food handling practices and consumption of undercooked meat. In many cases, the majority of pathogens contaminating carcasses at slaughter have been traced back to the farm of origin (8, 9). Thus, a snapshot of prevalent pathogenic organisms can be obtained by sampling farming environments, which might be a challenge when considering free-ranging wildlife species.

## The wildlife industry of Namibia

A greater economic weight currently placed on the wildlife meat industry and the tourism sector in Namibia compared to livestock farming has led to the establishment of numerous managed wildlife conservancies and to the spiralling of game farming units on many private farmlands, including some rural areas. In some areas, ruminant wildlife species is reared in close proximity to livestock which potentially results in a spill-over of infections in either direction. About 15–25% of private farmland is used for commercial game rearing, primarily for ranching, leisure hunting, live game capture and wildlife viewing (10). The Namibian population estimates of food-producing wildlife species other than fish and forest-dwelling invertebrates has been reported at a minimum of 2 million (11, 12). In terms of percentages, the major wildlife species under current and future consideration for commercial game meat export are: springbok (*Antidorcas marsupialis*), gemsbok (*Oryx gazella*), kudu (*Tragelaphus strepsiceros*), mountain zebra (*Equus zebra hartmannae*) and red hartebeest (*Alcelaphus buselaphus*) (11). These species provide the bulk of game meat with gemsbok, the kudu and springbok contributing approximately two-thirds of the total game meat produced on freehold farms. In 2009, Gemsbok and Springbuck population estimates stood at 388,411 and 731,563 respectively (11). Between 2001 and 2005, approximately 4,300 tonnes of game meat were produced annually in Namibia (13). Recent years have witnessed a huge increase in the production and export of wildlife meat to Europe and South Africa to meet the demands of discerning, affluent consumers who prefer meat produced in a sustainable and eco-friendly environment, where ethical and safe harvesting methods safeguard wholesomeness and nutritional value (12, 14). Between 16,000 and 26,000 tonnes of game meat is produced annually in Namibian farmlands for regional and international export markets, local supply and for personal consumption. This also includes meat that is produced during trophy hunting (15). Indeed, 275 tonnes of game meat were exported to South Africa and the European Union between 2009 and 2011 (as recorded by registered game export establishments). In this year's hunting season (April to August 2012), an average of 86 tonnes of game meat and game meat products are being exported monthly to South Africa, illustrating a booming wildlife meat industry in Namibia. Concurrent with the expansion of wildlife in Namibia is a decline in free range domestic animal farming, particularly sheep and cattle, resulting in some export abattoirs processing game meat during the wildlife hunting season using their under-utilised processing facilities (16). A clear worldwide demand for meat from species such as springbok, gemsbok, blesbok, eland, wildebeest and kudu exists and it is anticipated that this will continue to increase (14, 17, 18).

## Potential implications of zoonoses to the wildlife industry

The impact of zoonoses on human and animal health and welfare cannot be emphasised enough. The growing world population requires more food, especially safe and wholesome sources of protein. As a result, food security and safety issues have taken centre-stage on the global platform – all geared to safeguard human health. Since some zoonoses are notifiable diseases, these consequently can impose a huge economic burden on farmers through compulsory slaughter, resulting in loss of access to export markets and the local meat industry. With ruminant wildlife species increasingly entering the human food chain, coupled with a thriving managed wildlife for tourism purposes in Namibia, it is prudent to examine the extent to which such diseases may affect this emerging industry.

Around 60% of all human pathogens are zoonoses that are equally harboured by domestic and wild animals (19). And of the emerging infectious diseases, 75% of these are zoonoses predominantly associated with wildlife animals (4), thus clearly highlighting an increasing threat arising from these animal species. As outlined above, Namibia's thriving wildlife population is reared and slaughtered for both domestic and export markets, placing wildlife on a different platform from its traditional past. Because rearing and slaughter practices tend to differ from those of livestock species, concerns on meat safety have risen and inevitably these have presented a challenge to the existing regulatory framework of the meat industry. The aim of this article is to examine the potential zoonotic risks from wildlife and reared animals in Namibia as confirmed by laboratory investigations and documented literature findings during 1990–2009 to provide the basis for initiating a multi-disciplinary evidence-based approach to control such diseases.

## Data collection

The criteria for including zoonotic zoonoses in this manuscript were based on laboratory investigations during 1990–2009 and literature findings. Factors such as potential public health importance, morbidity, mortality and disease burden in both domestic and wild animals were also considered. Information on food-borne- and non-food-related zoonoses was extracted from annual surveillance reports of the Directorate of Veterinary Services in Namibia generated between 1990 and 2009 – primarily by their Central Veterinary Laboratory (CVL). All notifiable diseases were confirmed by an appropriate laboratory test. The major notifiable diseases in Namibia include rabies, Rift Valley fever (RVF), anthrax, brucellosis, chlamydiosis (psittacosis), Johne's disease, bovine tuberculosis and selected exotic diseases (20). Further confirmation of notifiable diseases is obtained from regional or international reference laboratories, including the Onderstepoort Veterinary Institute (South Africa) and Instituto Zooprofilattico Sperimentale della Italia in Teramo (Italy). Similarly, in cases of inconclusive results or when an approved diagnostic test is unavailable, samples are forwarded to the regional OIE reference laboratory in South Africa. The monitoring of salmonellosis, pathogenic *Escherichia coli*, campylobacteriosis and *Clostridium perfringens* infections is a requirement under food law procedural notices of Namibia (21, 22). The Namibia Institute of Pathology (NIP), which provides laboratory services to the health delivery sector confirming all human cases, works closely with the National Institute of Communicable Diseases (NICD), a regional centre of excellence in infectious diseases for the African continent at large. Although the majority of diseases required laboratory confirmation, the diagnoses of echinococcosis, cysticercosis, dermatophytosis and sarcocystosis during the period under review were primarily based on clinical and pathological findings. For convenience, we classified these diseases according to the aetiological agent, highlighting those encountered at a higher frequency in wildlife species in each category.

## Viral diseases

### Rabies

Rabies is caused by a *Lyssavirus* belonging to the family *Rhabdoviridae* that targets the central nervous system (CNS) resulting in a rapidly progressive and fatal encephalomyelitis. Transmission from animals to humans is primarily through bites and contact with secretions–although bats have been implicated in relatively few cases (23, 24). Vampire bats are the reservoir hosts of rabies in the West Indies and some South American countries, but these have not been reported in Namibia. Rabies is notifiable in Namibia and has the widest geographical, domestic and wildlife species distribution in the entire Southern Africa Development Community (SADC) region, making it a serious threat to the public, in particular farmers and hunters. Increased sporadic outbreaks of rabies in dogs, bovines and wildlife are common in northern and central regions of Namibia where up to 96% of deaths have been reported in the kudu (*Tragelaphus strepsiceros*), jackal (*Canis mesomelas*), bat-eared fox (*Otocyon megalotis*) and lion (*Panthera leo*) (23, 25, 26). The jackal is the recognised reservoir of rabies in southern Africa which has been implicated in unusual epizootics in the kudu (*Tragelaphus strepsiceros*) with lions subsequently contracting the disease from the rabid kudu (23, 27, 28). Of particular significance, the seasonal variation of rabies has been linked to occurrences in dogs and domestic ruminants together with the seasonal reproductive pattern of black-backed jackals (29). This epidemiological pattern is yet to be fully exploited in the control of rabies. Current control strategies centre on the restriction of animal movements and compulsory vaccination of dogs. This is supported by legislation which stipulates the need for a valid permit for all animal movements and mandatory current vaccination record for dogs. Fortunately, rabies vaccination is free for dogs, cats and other carnivores in Namibia, thereby removing the cost barrier from all pet owners. Despite this, the species distribution of rabies between 1990 and 2009 in Namibia was widespread (Table 1). Not surprisingly, majority of rabies cases were recorded in communal areas where huge numbers of dogs freely roam leading to a rapid spread through uncontrolled bites and contact with infected secretions. Furthermore, compliance with vaccination is relatively poor owing to lack of awareness on the risks of rabies in the rural communities. As such, there is a need for the regulatory authorities to re-evaluate the effectiveness of current approaches to controlling rabies in Namibia. Initiatives are also required for controlling the disease in wildlife apart from movement restrictions and monitoring. The kudu and the jackal had the highest prevalence of rabies, indicating the importance of the jackal in the transmission of rabies to wild ruminants. Considering the kudu as a food-producing wild animal, it would be prudent to prescribe a precautionary approach in harvesting this species. The need to devise novel approaches in controlling rabies in the wildlife population should therefore be a priority.

**Table 1:** Distribution of confirmed cases of rabies across species between year 1990 and 2009 in Namibia

Species/animal type	Number affected
Dog	1557
Cattle	1439
Kudu	429
Jackal	351
Goat	278
Cat	189
Sheep	89
Bat-eared fox	49
Fauna (unknown)	45
Horse	28
Mongoose	26
Donkey	24
Honey badger	15
Mouse	14
Hyaena	13
Eland	12
Suricate	12
Pig	8
Gemsbok	7
Cheetah	6
Oryx	5
Squirrel	5
Antelope	4
AARD wolf	4
Eland	3
Giraffe	2
Monkey	2
Baboon	2
Warthog	2
Duiker	3
Springbok	1
Rat	1
Hartebeest	1
Skunk	1
Wildebeest	1
Lion	1

Source: Ref. [26].

**Table 2:** Recorded cases of tetanus and botulism in farm animals and ostriches between 1990 and 2009 in Namibia

Species/animal type	Number affected with tetanus	Number affected with botulism
Cattle	53	1403
Sheep	46	118
Goat	33	113
Pig	0	1
Duck	0	9
Ostrich	0	4
Chicken	0	2
Horse	0	1
Bird	0	2
Suricate	0	1

Source: Ref. [26].

## Rift Valley fever

Rift Valley fever (RVF) is an arthropod-borne disease in mammals and humans caused by a Phlebovirus of the *Bunyaviridae* family (30). The disease is widespread in the southern African region, having been reported in South Africa, Namibia, Madagascar, Zimbabwe and Botswana in the last 3 years (31, 32). Lack of a well-established wild reservoir host complicates the epidemiology of this disease. Direct transmission from infected ruminants to naive hosts has been linked to *Aedes* spp and also a large number of potential vectors with a different bio-ecology (33). Nevertheless, the diagnosis of RVF was primarily based on serology which led to confirmation of the disease in humans, rhinoceros (*Diceros bicornis*) and lion (*Panthera leo*), including sheep and goats predisposed by a high level of parasitism (26, 34). Elsewhere, RVF has been reported in other wildlife species further suggesting the importance of this disease. In Kenya, 71.4% of buffaloes were positive for neutralising antibodies in Ijara district, 50% in Nakuru district, 37.5 % in Nairobi and Laikipia districts and 22.6% in Tana River districts, suggesting buffaloes to be a highly susceptible host. High levels of RVF viral antibodies were also reported in the Zimbabwean black rhino (*Diceros bicornis*), white rhino (*Ceratotherium simum*), buffalo, waterbuck (*Kobus ellipsiprymnus*) and the Kruger National Park African buffalo (35–37). Although the likelihood of a spill over of infection from either animal species exists, the author was unable to detect the virus by PCR in springbok (*n*=112) reared in close proximity to domestic animals with a history of RVF outbreaks. Direct exposure to infected animal secretions is associated with human mortality from RVF infection. In Namibia, periodic outbreaks of RVF often result in significant losses of livestock but the potential dangers of the disease to humans have only been realised in the past few years, necessitating a compulsory vaccination in all high risk areas (26). This exercise has been facilitated by the provision of free vaccination by the State to all high risk farms, particularly poor farmers in marginalised areas. As a result, every farmer is required to provide evidence of vaccination to a state veterinarian upon demand to ascertain compliance. Whilst implementation of such vaccination programmes appears relatively straightforward in domestic animals, application of similar programmes in free-ranging wildlife animals may be an insurmountable task. With the possibility of a spillover from domestic to wild animals and vice versa still existing, there is a need to explore the implementation of novel effective control strategies in both species.

### Crimean-Congo hemorrhagic fever

Crimean-Congo hemorrhagic fever (CCHF) caused by a Nairovirus of *Bunyaviridae* family (38) inflicts a high mortality rate in humans (39). The virus is transmitted by Argasid or Ixodid ticks belonging to the genus *Hyalomma* and the worldwide distribution closely matches that of its main arthropod vector (40). CCHF is a public health concern worldwide due to its high fatality rate and also its preponderance to be transmitted nosocomially (40, 41). Ticks acquire CCHFV when feeding on viraemic animals (42) with small vertebrates, such as hares and hedgehogs, acting as amplifying hosts (43). The disease has been reported in a wide variety of wild animals; giraffe (*Giraffa camelopardalis*), rhinoceros (*Ceratotherium simum* and *Diceros bicornis*), eland (*Taurotragus oryx*), buffalo (*Syncerus caffer*), Burchell's zebra (*Equus burchelli*), gemsbok (*Oryx gazella*) and greater kudu (*Tragelaphus strepsiceros*), wild carnivores, primates, smaller antelopes, pigs, blue wildebeest (*Connochaetes taurinus*) and ostriches (44, 45). In humans, the disease is primarily an occupational hazard being reported more in shepherds, abattoir and leather factory workers, veterinary doctors, farmers, health personnel, soldiers, peasants, forest workers and hunters but rarely in picnic-goers and people involved in open-space activities (39, 40, 46). In Namibia, relatively few cases of CCHF have been reported in livestock farmers, but there is a lack of comprehensive or detailed investigation into the prevalence of the disease and its transmission pathways to humans in affected farming communities. Despite this, the wide host spectrum makes CCHF a major threat to humans, domestic and wildlife animals alike and, therefore, calls for an urgent review of current precautionary and control approaches.

## Bacterial diseases

### Anthrax

**Fig. 1:**
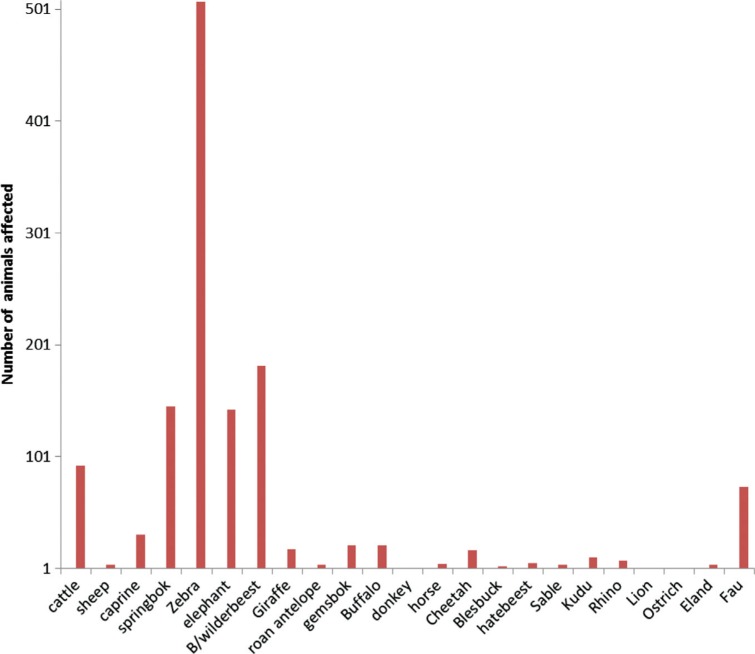
Species distribution of anthrax cases across domestic and wildlife recorded between year 1990 and 2009 in Namibia (single numbered animals are not visible on the graph. Source: Ref. [26].

Anthrax is caused by a Gram-positive, sporulating bacterium, *Bacillus anthracis* and primarily affects all warm blooded animals, including herbivorous livestock and wildlife species (47). After rabies, anthrax has the second highest number of cases and widest geographical, domestic and wildlife species distribution in Namibia (Fig. 1). Transmission is usually via direct contact with infected material, inhalation of spores, ingestion of contaminated feed or water and, in rare cases, via blood-sucking insects. Characteristically, animals are often found dead due to overwhelming bacteraemia and toxaemia. During this phase, the bacterium exists in a vegetative form which quickly sporulates upon exposure to air resulting in resistant spores that can survive in the soil for up to 50 years. Due to this, post-mortem examination of anthrax cases is contra-indicated and carcasses are usually incinerated or buried whole. In Namibia, this is a notifiable disease and control is enforced by a regulatory requirement for annual vaccinations for all cattle with voluntary vaccination extended to sheep and goats. Furthermore, imported cattle older than 3 months are vaccinated every 12 months. We presume this to contribute significantly to the relatively low incidences observed in domestic animals compared to wildlife species (Fig. 1). Whilst implementation of such control practices is feasible in livestock species, there is a need to re-think on the modalities of applying these strategies in free-ranging wildlife species. Carcasses, resulting from peracute infection, are often detected after scavenger activity that litters the environment with spores, making it difficult to contain the infection (48). Moreover, contamination of environment with spores significantly contributes to perpetuation of the infection within the wildlife environment and therefore the population. Relatively high sero-prevalence rates among carnivores and to a lesser extent herbivores were reported, implying a regular non-fatal exposure in these wild animal species. Indeed, anthrax is endemic in Etosha National Park of Namibia, where fatal cases have frequently been detected in red hartebeest (*Alcelaphus buselaphus*), wildebeest (*Connochaetes taurinus*), zebra (*Equus burchellii*), springbok (*Antidorcas marsupialis*), eland (*Tragelaphus oryx*), giraffe (*Giraffa camelopardalis*), ostrich (*Stuthio camelus*), white-backed vulture (*Gyps africanus*), chacma baboon (*Papiour sinus*) and polecat (*Ictonyx striatus*) (39, 40, 49). Of the herbivores, zebra (*Equus burchelli*), elephant (*Loxodonta africana*), wildebeest (*Connochaetes taurinus*), springbok (*Antidorcas marsupialis*) and buffalo contributed 97% of the cases in the period under review (25). Elsewhere, anthrax has been reported in wildlife in the Serengeti (Tanzania) (50) and the Kruger National Park (South Africa; 51). The detection of *B. anthracis* in 3.3% of the water samples and 3% of soil samples collected at different times of the year from 23 sites not associated with anthrax, confirmed persistent environmental contamination with bacterial spores (52). High prevalence rates (>50%) were found in faeces of scavengers (vulture, jackal and hyaena) collected within the vicinity of confirmed anthrax cases and 26% of water samples from associated national park waterholes were positive for anthrax organisms (52). Predation is a common method of transmitting anthrax to carnivores as seen in the cheetah (*Acinonyx jubatus*) whose source of infection is often the baboon (*Papio ursinus*) or red hartebeest (*Alcelaphus buselaphus*) (53, 54). On the contrary, exposure patterns in dogs have reflected known patterns of endemicity and in some cases provided new information about anthrax in the ecosystem, suggesting the potential of carnivores to be indicators of the disease (55). While species-specific ecological factors may affect anthrax exposure processes in Namibia, transmission cycles in different host species are still highly interrelated. Apart from restricting domestic animal movements within the national parks, there seems to be a lack of an effective strategy to control anthrax in the wildlife population, therefore calling for an exploration of new intervention approaches across southern Africa.

### Brucellosis

Brucellosis in Bovine, Ovine and Caprine species has been reported at low incidences in Namibia (26). To date, no seropositive cases have been reported in free-ranging species of wild animals in Namibia. We recently screened 900 springbok (*Antidorcas marsupialis*) from 29 mixed farming units and found no serological evidence of brucellosis in these species (56). *Brucella spp* have been reported in the African buffalo (*Syncerus caffer*), mountain zebra (*Equus zebra*), waterbuck (*Kobus ellipsiprymnus*), hippopotamus (*Hippopotamus amphibious*), puku antelope (*Kobus vardonii*), wildebeest (*Connochaetes taurinus*), buffalo (*Syncerus caffer*), feral pigs, bushbuck (*Tragelaphus scriptus*), Burchell's zebra (*Equus burchelli*), impala (*Aepyceros melampus*), eland antelope (*Taurotragus oryx*) and other wildlife species in SADC countries and elsewhere (57–60). Sero-prevalences of 23 and 48% were reported in African buffalo populations in The Kruger National Park and Zimbabwe, respectively, in the absence of direct contact with domestic animals (57). Although *B*. *melitensis* has been rarely reported in wildlife, it is endemic in the Kafue lechwe (*Kobus leche spp kafuensis*) reared at the wildlife–domestic animal interface in Zambia (61). Isolated cases have also been reported in *Rupicapura rupicapra* and *Capra ibex* in Europe (62). The incidence of brucellosis in domestic animals is often governed by the transmission dynamics (epidemiology), especially in extensive production systems where large herds may offer optimal conditions (60).

**Fig. 2:**
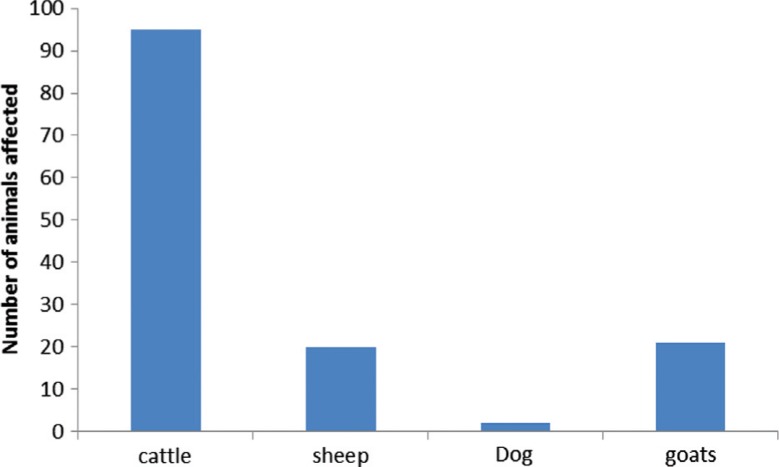
Distribution of brucellosis across domestic species confirmed between year 1990 and 2009 in Namibia. Source: Ref. [26].

However, it is unclear why a relatively higher incidence was obtained in cattle than other domestic animals (Fig. 2). There is a regulatory requirement for all heifers aged 3–11 months to be vaccinated against brucellosis, whereas sheep and goats are vaccinated on a voluntary basis. In addition, all imported animals are pre-screened against brucellosis prior to and upon arrival. Brucellosis is one of the highly regulated diseases in Namibia where millions of dollars are spent annually on surveillance in both wildlife and domestic animals. Vaccination coverage data in small stock is currently unknown and farmers receive an automatic on-farm *Brucella* status upon generation of a movement permit from the Namibia Livestock Identification and Traceability System (NAM-LITS). In very few isolated caprine cases, the disease has been confirmed by bacterial isolation. On the other hand, the use of serological tests has been frequently complicated by the specificity of the tests, particularly arising from the effect of uncontrolled vaccinations with Rev.1 vaccine strain. The Complement Fixation Test (CFT) and Rose Bengal Test (RBT), in particular, have low specificity when testing sera from small ruminants vaccinated subcutaneously with Rev.1 strain, which can be improved when the vaccine is administered via the conjunctival route (63). The possibility of false positive serological reactions (FPSR) due to sharing of antigenic determinants with other Gram-negative bacteria, especially *Y. enterocolitica* O: 9 (64), should always be considered in all sero-positive cases.

### Salmonellosis

**Fig. 3:**
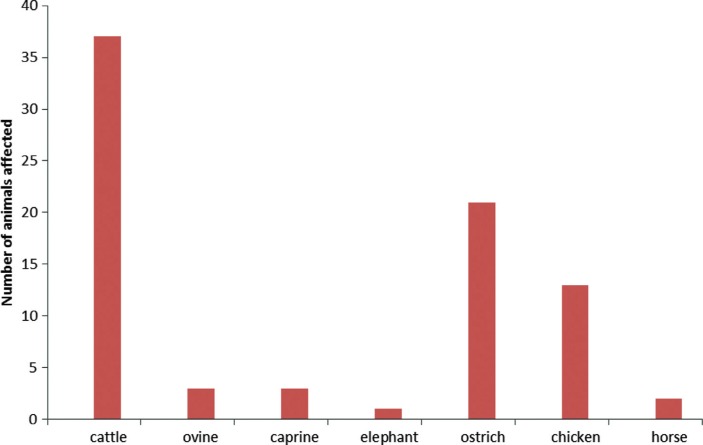
Confirmed cases of salmonellosis in different animal species recorded between year 1990 and 2009 in Namibia. Source: Ref. [26].

Non-typhoidal salmonellosis represents an important human and animal disease of worldwide importance. There are over 2,000 *Salmonella* serovars, which all have the potential to cause illnesses in humans and animals despite certain serovars (*S*. Typhimurium, *S*. Enteritidis and *S*. Newport) being commonly implicated in human infections and *S*. Dublin and *S*. Onderstepoort more commonly associated with cattle and sheep, respectively, in South Africa (65–67). It is interesting to note that some serovars are associated with infections in crocodiles (*Crocodylidae*) (44). Forty-five *Salmonella* serovars were isolated from fish meal, fresh red meat (beef and lamb), cattle feedlot, and meat and bone meal samples in Namibia. A wide distribution of salmonellosis cases was observed in Namibia (Fig. 3), with cattle and ostriches topping the list. Although a few cases were recorded in elephants, the role in transmission could be associated with contamination of the environment and water sources.

### Campylobacteriosis

**Fig. 4:**
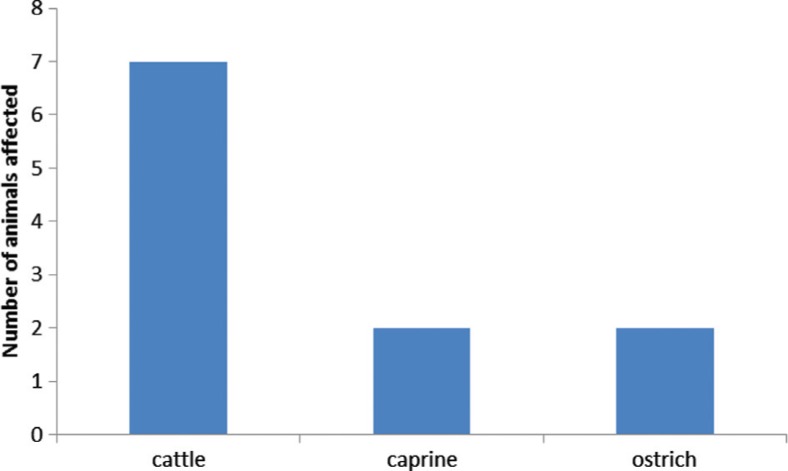
Confirmed cases of camplylobacteriosis across species recorded between 1990 and 2009 in Namibia. Source: Ref. [26].

*Campylobacter spp* cause one of the most common bacterial food-borne infections in some parts of the world (68, 69). The bacteria normally inhabits the intestinal tract of warm-blooded animals, such as poultry and cattle, and are frequently detected in foods derived from these animals (70). Despite 60–80% of cases of campylobacteriosis being attributed to chicken (69), none has been recorded in chickens in Namibia (Fig. 4), mainly because *Campylobacter* spp rarely cause pathology in this species, often regarded as a commensal that causes severe gastro-enteritis in humans. In particular, *C. jejuni* primarily colonises the chicken gut and is excreted in high numbers in faeces without adverse symptoms (69). Campylobacteriosis in sheep and goats, caused by *Campylobacter fetus* ssp. *fetus intestinalis*, is of less zoonotic importance compared to *C. jejuni* and *C. coli* (71, 72). Although the prevalence of *C. jejuni* and *C. coli* in sheep at slaughter was reported to be 44 and 17%, respectively, compared to 24% in cattle and 94% in pigs, there is little evidence to suggest that red meat and wild game species present a risk to human infections (71). Relatively little work has been done on zoonotic *Campylobacter* spp in Namibia as reflected by thevery few cases recorded (Fig. 4).

## Escherichia coli *infections*

Pathogenic *Escherichia coli* strains have been a major concern in the meat industry for decades (60). Among these, Shiga toxin-producing *E. scherichia coli* (STEC) which are harboured in the intestinal tracts of ruminants still present a major public health concern. Although STEC O157:H7 was associated with several outbreaks since its first report in 1983, non-O157 strains are increasingly being encountered in recent years, now accounting for 20–70% of STEC infections throughout the world. Of the 81 different non-O157 serogroups associated with shiga toxins’ presence, 71% of these were represented by six serogroups; O26, O45, O111, O103, O121 and O145 (73–76). Direct or indirect contact with ruminant faeces is the leading antecedent to STEC infections in humans (77). Principal virulence factors associated with these strains are either plasmid borne or phage encoded, increasing the likelihood of a spontaneous horizontal gene transfer between species or serotypes. Moreover, the occurrence of stx-phages as free infectious particles in the environment and their persistence in water systems contribute to a high dispersal rate of these virulence genes (78). There is relatively very little work done on STEC in Namibia and factors affecting meat safety policy include lack of laboratories offering *E. coli* serotyping, insufficient risk assessment data and lack of updated food laws. Performance standards of slaughtering process of both domestic and wildlife species are usually based on *Enterobacteriaceae*, generic *E. coli* and generic *Salmonella* levels (79). Although not confirmed by the culture method, *E. coli* O157H7 had previously been isolated in eight out of 95 meat samples using Polymerase chain reaction (PCR) in Namibia. Prompted by high *Enterobacteriaceae* counts in five batches of springbok meat, we undertook a limited survey to determine the prevalence of STEC in rectal contents and found none (80). As such, a comprehensive survey is therefore required to define the role of wildlife ruminants in the epidemiology of STEC to inform on aspects of meat safety.

### Chlamydiosis

**Fig. 5:**
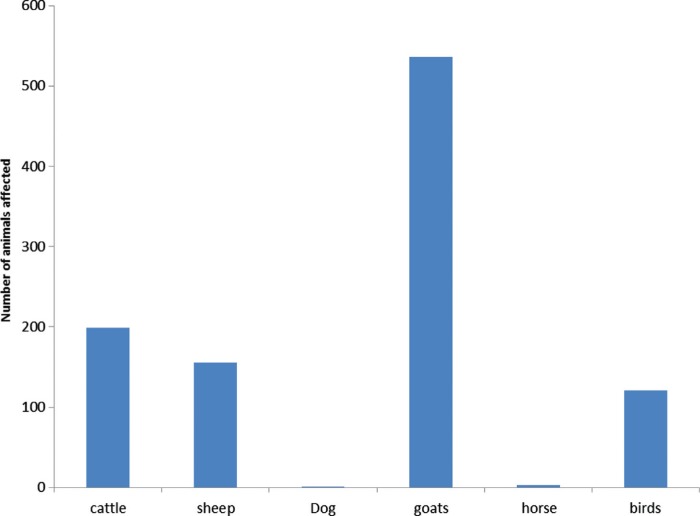
Cases of chlamydiosis and/or psittacosis in domestic species recorded between year 1990 and 2009. Source: Ref. [26].

*Chlamydiaceae* are Gram-negative obligate intracellular bacteria that primarily replicate in mucosal epithelial cells of the conjunctivae, the respiratory, urogenital and gastrointestinal tract and are responsible for a broad range of diseases in animals and humans (81). The genus *Chlamydia* includes *C. trachomatis* (humans), *C. suis* (swine) and based on morphological characters and 16S rRNA and 23S rRNA gene sequences, the genus *Chlamydophila* includes *C. psittaci* (avian), *C. felis* (cat), *C. abortus* (sheep, goat and cattle), *C. caviae* (guinea-pig), the former species *C. pecorum* (sheep and cattle) and *C. pneumonia* (humans) (32, 82). Previous work in Namibia indicated that *C. abortus* infections were prevalent in all the geographical regions tested with on–farm and individual sero-prevalence levels in goats ranging between 8 and 50% (83, 84). Among the domestic animals, goats were over-represented (Fig. 5). In wild ungulates, sero-prevalence rates ranging from 23 to 60% were reported in European wild boar, red deer, fallow deer, roe deer, mouflon, barbary sheep, Southern chamois and Iberian ibex (85). Chlamydiosis is also widespread in Nile crocodiles (*Crocodylus niloticus*) on Zimbabwean farms although the danger of zoonotic transfer is not yet known (44). The possibility of transmission from livestock to free-living bird species has been confirmed using selected species: *Chlamydia suis*, *Chlamydia muridarum* and *Chlamydophila abortus* (86). Although no conclusive data on the prevalence and relevance of chlamydiae in wild mammals are available in Namibia, there are a few unreported studies on the prevalence of *Chlamydophila abortus* in targeted wildlife species. More studies are therefore required to provide the basis of implementing effective control strategies. Nevertheless, in-depth integrated intervention strategies can also be carried out based on available data of *C. psittaci* and *C. abortus* in domestic animals, as shown in Fig. 5.

## Clostridial infections

*Clostridium perfringens* type A and *Clostridium difficile* food poisoning are among the most commonly identified and hypothesized food-borne illnesses typically transmitted via improper handling of contaminated foods (http://www.ncbi.nlm.nih.gov/pubmed/20642351). Type C of *Clostridium botulinum* intoxications have been reported in 117 species of feral birds and a wide variety of mammalian species (http://www.merckvetmanual.com/mvm/index.jsp?cfile=htm/bc/205400.htm). Vaccination of domestic animals especially cattle, sheep and goats against clostridial diseases is one of the most extensively practiced preventative measure in Namibian farming community. Despite the vaccinations, cases of *Cl. Tetani* and *C. botulinum* were recorded every year (Table 2) possibly resulting from insufficient vaccination coverage, vaccine failure or seasonal mineral deficiency (like phosphate) in grazing environments. To date, no cases of *C. difficile* have been recorded in Namibia.

## Parasitic diseases

### Echinococcosis

*Echinococcus granulosus* is a tapeworm with nine genotypes which inhabits the small intestine of canines, whereas *E. multilocularis* infects foxes, raccoons, small rodents (definitive hosts) with dogs and cats acting as accidental hosts (69). Adult *E. granulosus* worms occur in large numbers in the small intestine of many wild animals, including hyaena, lion, wild dog (*Lycaonpictus*) and leopard. Echinococcosis is commonly encountered in free-ranging Burchell's zebra, buffalo, greater kudu, and hippopotamus, and impala may serve as intermediate hosts (44). In Namibia, hydatid cyst of *Echinococcus granulosus* has been reported in giraffes (*Giraffa camelopardalis*) (87) and is commonly reported in livestock species.

### Trichinellosis

*Trichinella* is one of the world's most widely distributed food-borne zoonotic nematode of mammals, birds and reptiles (88). Natural infection with *Trichinella* spp. has been described in more than 150 mammalian species (89) but there are few reports in wildlife species emanating from Africa. Noteworthy, *Trichinella nelsoni* and *T. spiralis* were found in wild game in South Africa (20) whilst *T. zimbabwensis* was first described in southern African monitor lizards (*Varanus niloticus*) and Nile crocodiles (88, 90). With a wide species distribution, including monitor lizards, wild pigs, domestic pigs, wild zebra and crocodiles, the risk of transmission is relatively high. Although, the incidences of *Trichinella* spp. in domestic and/or wildlife is still limited to Namibia, *Trichinella* genotypeT8 has been detected in a lion and larvae have been found in a spotted hyena and a black-backed jackal from Etosha National Park (88). The prevalence of *T. zimbabwensis* in Namibian wild crocodiles and in their predators remains unknown; however, the natural distribution of *T. zimbabwensis* in South African wild crocodiles includes all the major river systems in the Kruger National Park where *T. zimbabwensis* occurs in 10 out of every 12 culled crocodiles (91). Recent years have witnessed a rapid expansion of the crocodile industry in Namibia and some of these are often preyed upon by leopards (92).

### Cysticercosis

Worldwide, human cysticercosis is a major public health concern with *T. saginata* and *T. solium* being frequently associated with cattle and swine, respectively (93, 94). The parasite is common in wildlife and domestic species (95). Clinical diagnosis is often difficult and confirmation is usually at post-mortem, suggesting this disease could possibly be under-diagnosed. In South Africa, cysticercosis appears to be most prevalent in the Eastern Cape Province, where pigs roam freely and sanitation facilities are inadequate or non-existent (20). Segments of tapeworms often feature as an ingredient of concoctions prepared by traditional healers and are suspected to be sources of many of the cases of cysticercosis in South Africa (20, 96). In Namibia, 865 bovine and five sheep carcasses were detained for cysticerci during meat inspection at six export abattoirs. Infestation has also been reported in impala, buffalo and blue wildebeest (36). Despite the high prevalence (97) of cysticercosis in Namibian livestock, the disease is not regarded as reportable, hence data on the burden in humans is lacking for most regions. Preventative approaches against taeniasis and cysticercosis need to be re-evaluated, especially at primary production level, that is, communal and commercial farming areas. A holistic approach involving key sectors like the Ministry of Health and Social Services is also required to improve early detection and control of this disease.

## Rickettsial diseases

### Coxiella burnetti (Q fever)

Q fever is a tick-transmitted zoonosis caused by *Coxiella burnetii* (98) with a worldwide distribution and a potential to infect most vertebrate species, including humans, ruminants, rodents, cats and reptiles (69). The traditional reservoirs of infection are domestic animals: cattle, sheep and goats, with parturient domestic cats and dogs implicated as sources of outbreaks (99). In Namibia, most cases were recorded in caprine species, but there is lack of documented evidence of this infection in wild animals. As such, the potential risk to public health remains highly likely but is unknown.

## Fungal diseases

### Dermatophytosis (Ringworm)

Ringworm is caused by different species of dermatophytes with the zoophilic species: *Microsporum canis*, *Trichophyton verrucosum* and *T. mentagrophytes* predominantly associated with wild and domestic animals depending on the geographic region (100). Ringworm is routinely diagnosed in Namibian cattle; however, to date no cases have been reported for wildlife.

## Other bacterial and parasitic diseases

**Fig. 6:**
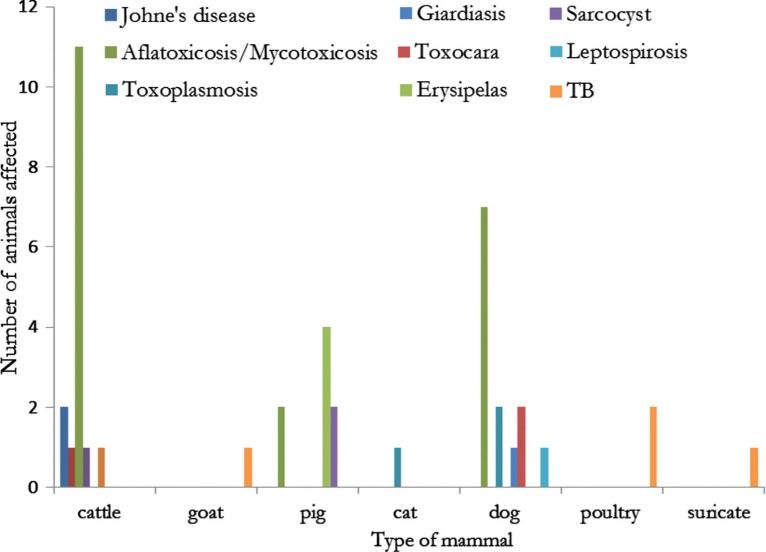
Species distribution of other zoonotic infections recorded in both domestic and wild animals between 1990 and 2009 in Namibia. Source: Ref. [26].

Many diseases tend to pose a zoonotic threat directly or indirectly through consumption of livestock products, including wildlife (Fig. 6). Importantly, sarcocystosis is a zoonotic disease caused by *Sarcocystis spp*, one of the most commonly found parasites in domestic animals worldwide (101). It is a cyst-forming coccidian parasite with obligatory two host life cycles involving carnivores as definitive hosts and herbivores or omnivores as intermediate hosts (102). The cysts have been reported in many domestic and wild animals, including rat, moon rat, bandicoot, slow loris, buffalo, monkey and man. The known definitive hosts for some species of *Sarcocystis* are: the domestic cat, dog and the reticulated python (103–106). Although a few cases have been reported in Namibian in sheep and lion (*Panthera leo*) at post-mortem, the infection is probably under-diagnosed (26, 107).

Johne's disease is caused by *Mycobacterium avium subsp. paratuberculosis* (MAP) (108). Recently, this bacterium has received an increasingly wide interest because of a rapidly growing body of scientific evidence which suggests a possible link between MAP and Crohn's disease, a human inflammatory bowel disease (109). MAP is a potential human food-borne pathogen which is excreted in milk and survives current pasteurisation treatments (110, 111). Though a potential of wide host species exist, only a few cases were recorded in Namibian cattle.

Leptospirosis is an infectious zoonotic disease caused by spirochetal pathogenic *Leptospira species* of which *L. icterohaemorrhagiae* is the main serovar responsible for human disease (112). A variety of wildlife, including raccoons, white tailed deer, striped skunks, opossums, and red and grey foxes have been shown to harbour some *Leptospira* serovar*s* (113) but very few cases have been recorded in Namibia.

*Toxoplasma gondii* is a protozoan parasite affecting all warm-blooded hosts worldwide (114). Relatively, little is known about the prevalence of *T. gondii* in Africa although a prevalence rate of 9% in the San (Bushmen) people of Namibia and Botswana compared to 30% in the Indian and black communities of Kwazulu-Natal province of South Africa have been reported (20, 115). The parasite multiplies in the gastrointestinal tract of cats. Other mammals like captive carnivores, captive herbivores, free-living carnivores, free-living herbivores, moose (*Alces alces*), black bears (*Ursus americanus*), caribou (*Rangifer tarandus*), wolves (*Canis lupus*), mice (*Musmusculus* and *Peromyscus spp*), rats (*Rattus norvegicus* and *Sigmodon hispidus*), squirrels (*Sciurus spp*), rabbits (*Sylvilagus floridanus*), muskrats (*Ondatra zibethicus*), red foxes (*Vulpes fulva*), mink (*Mustela vison*), Burchell's zebra, (*Equus burchelli*), hippopotamus (*Hippopotamus amphibius*), African elephant (*Loxodonta africana*), sand gazelle (*Gazella subgutturosa marica*), mountain gazelle (*Gazella gazelle*), the Dorcas gazelle (*Gazella dorcus*), defassa waterbuck (*Kobus defassa*), lion (*Panthera leo*) and rock hyrax (*Procavia capensis*) can become infected by ingesting food or water contaminated with oocysts (116–120).

Of the 27 species belonging to the genus *Toxocara*, the majority infect carnivores from the *Canidae, Felidae, Viverridae, Procyonidae, Mustelidae* and *Herpestidae* families (121). *Toxocara vitulorum* is mainly found in tropical and subtropical regions, where it is a common parasite affecting water buffalo, Zebu cattle, lions and spotted hyenas (*Crocuta crocuta*) and is considered a low-level zoonotic disease agent (122, 123). *T. canis*, *T. cati* and *T. pteropodis* are the aetiological agents of human toxocariasis (121) but these have not yet been recorded in Namibia.

*Mycobacterium bovis* infects a wide range of hosts, including domestic livestock, wildlife and humans, and it has been reported in cattle, insectivores, rodents, feral Asian water buffalo (*Bubalus bubalis*), white tailed deer (*Odocoileus virginianus*), brushtail possum (*Trichosurus vulpecula*), ferret (*Mustela furo*), feral wild boar (*Sus scrofa*), African buffalos (*Syncerus caffer*), lions and lechwe (*Kobus leche*) (49–51, 123). The control of bovine tuberculosis and atypical mycobacterioses in developing countries is difficult because of the existence of wildlife reservoirs (50, 124). Over the past 20 years, no cases have been reported in Namibian cattle although insignificant numbers have been recorded in poultry and suricate. This is probably due to the effectiveness of the bovine tuberculosis eradication programme implemented by the Namibian regulatory authorities and the subsequent routine monitoring and testing of all dairy farms and imported animals before and when they enter the country.

Oocysts of *Cryptosporidium* and cysts of *Giardia* occur in the aquatic environment throughout the world. They have been found in most surface waters, where their concentration is related to the level of faecal pollution or human use of the water (125). The sporadic high counts of *Giardia* and *Cryptosporidium* in the Namibian raw water sources indicate that a multiple-barrier approach must be followed to ensure the safe operation of treatment plants using polluted source water (126).The role of wildlife in transmitting *Giardia* to humans has been controversial although a variety of *Giardia* species has been isolated from wild mammals, birds, amphibians and reptiles (127).

Swine erysipelas (SE) is a zoonotic disease caused by the bacterium *Erysipelothrix rhusiopathiae* which also causes a serious disease in domestic pigs and wild boars (128) in Namibia. However, human erysipelas is rare and limited to animal handlers.

## Discussion and future perspectives

Illegal wildlife trade is estimated to be a multibillion-dollar business involving unlawful harvest and trade in live wildlife animals, thus imposing a significant threat of infection to humans and domestic and wild animals. Zoonotic diseases can potentially lead to severe poverty, hunger and a compromised global health through banned trade and travel restrictions. However, the economic impact and the extent to which zoonoses contribute to human illnesses and death in the Namibian population are unknown. Though no outbreaks have been reported in Namibia, the emerging zoonoses avian influenza and swine influenza have been given significant attention at the expense of endemic zoonotic diseases. As such, prevalent zoonotic infectious diseases are likely to be considerably under-diagnosed because of the disproportionate focus on the disease of importance.

Economic importance for farmers or the meat industry is considered as one of the most influential factors in developing national strategies, plans and setting priorities on specific zoonotic diseases in Namibia. To protect the domestic food-producing animals and their export markets, brucellosis, Bovine spongiform encephalopathy (BSE), tuberculosis, anthrax, rabies, RVF and avian influenza have been given much financial and regulatory attention. Recently, significant amounts of resources have been channelled for the control of rabies in companion animals and RVF in domestic food-producing animals due to outbreaks of the two diseases. In the developing world, 56 zoonotic diseases are responsible for approximately 2.5 billion cases of human illness and 2.7 million deaths annually. Recent studies in Africa and beyond indicate the importance of the most common 13 zoonotic diseases in terms of their impact on human deaths and effect on livestock in the descending order: zoonotic gastrointestinal disease, leptospirosis, cysticercosis, zoonotic tuberculosis, rabies, leishmaniasis, brucellosis, echinococcosis, toxoplasmosis, Q fever, zoonotic trypanosomiasis, hepatitis E and anthrax (129). When information on the 13 most common zoonotic diseases in the developing world is applied to Namibia, evidence indicates a poorly defined national strategy and priority settings for nine of the diseases. In this regard, risk-based priority for monitoring and surveillance should be recommended for Namibia to facilitate directing efforts and resources to where they are most needed.

A wide spectrum of zoonotic diseases has been recorded primarily in domestic animal species and select wildlife species in Namibia over a 20-year period. With an increasing interaction between humans, domestic and wildlife, the threat of transmission of zoonotic diseases has never been so alarming. Wildlife species are well-documented reservoirs of many infectious diseases and evidence of transmission between domestic and such species exists. The preference of organically produced and highly nutritious game meat by today's population further increases the risk of food-borne zoonoses. Game farming has become thriving business in Namibia's arid regions, ranging from small-holder conservancies in rural areas to large commercial game ranches which slaughter for the export market. It is therefore imperative to examine the extent of this emerging/re-emerging threat posed by the rising interaction between humans and animals. By analysing such data, an evidence-based case can be formulated for a multidisciplinary approach to tackle such diseases within the framework of a ‘one health’ concept. It is worthy to appreciate that control of the zoonoses associated with food-producing domestic species can be implemented at any point along the food production continuum, but such a farm-to-fork approach may be difficult to apply in free-ranging wildlife species. Whilst this may be feasible for food-producing wild animals, this tends to be more difficult when dealing with wild carnivores.

Although, control of zoonoses may appear to be feasible in domestic species, evidence presented herewith shows a rising trend of certain diseases in domestic species, in particular rabies in the dog and cattle. However, the emergence of unusual and highly pathogenic serotypes or pathotypes as seen in the recent outbreak of haemorrhagic colitis and haemolytic uremic syndrome in Germany caused by enteroaggregative *E. coli* (130) calls for extra vigilance and improvement in diagnostic techniques. Furthermore, there is a need to devise novel surveillance strategies in wildlife since some diseases may not be clinically or pathologically discernible but serologically positive (131). Although there is a lack of data in humans, a clear need to re-evaluate existing control strategies and improve on monitoring and surveillance exists, especially on emerging zoonoses like avian influenza and swine influenza. In this respect, animals reared at the wildlife–livestock interface provide the best target group for assessing spill-over of infections which could potentially reach humans. Free-roaming domestic dogs and game species captured for translocation as well as those harvested for trophy and meat purposes may also be good indicator targets for monitoring and surveillance of public health risks.

A specialised reporting unit in the Directorate of Veterinary services for wildlife health, including foodborne related findings by role players should be established to create direct links between national game harvesting establishments and the different national game and game products’ inspection services. A reliable wild game traceability system should be developed at all harvesting levels in the country taking into consideration conservancies and large scale mixed game farms where only less than a third of farms have game fencing around their properties (15). Since wild rondents potentially interact with food producing wildlife, *Trypanosoma lewisi*, *Bacillus spp*, *Borrelia sp* and *Yersinia pestis* should be part of any future emerging zoonosis research needs in Namibia wildlife due to their detection and or presence of antibodies in rodents and shrews (132).

Also, challenges on fragmented meat safety legislation and lack of clear direction on implementation of the farm to fork concept in game industry should be addressed. In addition, the increasing worldwide threat of antimicrobial-resistant bacterial strains in particular methicillin-resistant *Staphylococcus aureus* (MRSA) and extended spectrum beta-lactamase (ESBLs) *Enterobacteriaceae* strains of animal origin require utmost attention. Thus, a multifaceted approach to control of each disease is required across all domestic and wild animals to curb transmission of such diseases to humans.
